# Molecular assays for antimalarial drug resistance surveillance: A target product profile

**DOI:** 10.1371/journal.pone.0204347

**Published:** 2018-09-20

**Authors:** Christian Nsanzabana, Frederic Ariey, Hans-Peter Beck, Xavier C. Ding, Edwin Kamau, Sanjeev Krishna, Eric Legrand, Naomi Lucchi, Olivo Miotto, Sidsel Nag, Harald Noedl, Cally Roper, Philip J. Rosenthal, Henk D. F. H. Schallig, Steve M. Taylor, Sarah K. Volkman, Iveth J. Gonzalez

**Affiliations:** 1 Foundation for Innovative New Diagnostics, Geneva, Switzerland; 2 INSERM 1016, Institut Cochin, Université Paris Descartes, Paris, France; 3 Service de Parasitologie-Mycologie, Hôpital Cochin, Paris, France; 4 Medical Parasitology and Infection Biology Department, Swiss Tropical and Public Health Institute, Basel, Switzerland; 5 University of Basel, Basel, Switzerland; 6 Walter Reed Army Institute of Research, Silver Spring, Maryland, United States of America; 7 Kenya Medical Research Institute/United States Army Medical Research Directorate-Kenya, Kisumu, Kenya; 8 Institute for Infection & Immunity, St George's University of London, London, United Kingdom; 9 Malaria Genetic and Resistance Group, Biology of Host-Parasite Interactions Unit, Institut Pasteur, Paris, France; 10 Malaria Branch, Division of Parasitic Diseases and Malaria, Centers for Disease Control and Prevention, Atlanta, Georgia, United States of America; 11 Mahidol-Oxford Research Unit, Faculty of Tropical Medicine, Mahidol University, Bangkok, Thailand; 12 Wellcome Sanger Institute, Hinxton, United Kingdom; 13 Big Data Institute, Li Ka Shing Centre for Health Information and Discovery, University of Oxford, Oxford, United Kingdom; 14 Centre for Medical Parasitology, Department of Immunology and Microbiology, University of Copenhagen, Copenhagen, Denmark; 15 Department of Infectious Disease, Copenhagen University Hospital, Copenhagen, Denmark; 16 Institute of Specific Prophylaxis and Tropical Medicine, Medical University of Vienna, Vienna, Austria; 17 Faculty of Infectious and Tropical Diseases, London School of Hygiene and Tropical Medicine, London, United Kingdom; 18 Department of Medicine, University of California, San Francisco, California, United States of America; 19 Amsterdam UMC, University of Amsterdam, Department of Medical Microbiology, Experimental Parasitology, Amsterdam, The Netherlands; 20 Division of Infectious Diseases and Duke Global Health Institute, Duke University Medical Center, Durham, North Carolina, United States of America; 21 Department of Immunology and Infectious Diseases, Harvard T. H. Chan School of Public Health, Boston, Massachusetts, United States of America; 22 Infectious Disease Initiative, Broad Institute of MIT and Harvard, Cambridge, Massachusetts, United States of America; 23 College of Natural, Behavioral, and Health Sciences, Simmons University, Boston, Massachusetts, United States of America; Arizona State University, UNITED STATES

## Abstract

Antimalarial drug resistance is a major constraint for malaria control and elimination efforts. Artemisinin-based combination therapy is now the mainstay for malaria treatment. However, delayed parasite clearance following treatment with artemisinin derivatives has now spread in the Greater Mekong Sub region and may emerge or spread to other malaria endemic regions. This spread is of great concern for malaria control programmes, as no alternatives to artemisinin-based combination therapies are expected to be available in the near future. There is a need to strengthen surveillance systems for early detection and response to the antimalarial drug resistance threat. Current surveillance is mainly done through therapeutic efficacy studies; however these studies are complex and both time- and resource-intensive. For multiple common antimalarials, parasite drug resistance has been correlated with specific genetic mutations, and the molecular markers associated with antimalarial drug resistance offer a simple and powerful tool to monitor the emergence and spread of resistant parasites. Different techniques to analyse molecular markers associated with antimalarial drug resistance are available, each with advantages and disadvantages. However, procedures are not adequately harmonized to facilitate comparisons between sites. Here we describe the target product profiles for tests to analyse molecular markers associated with antimalarial drug resistance, discuss how use of current techniques can be standardised, and identify the requirements for an ideal product that would allow malaria endemic countries to provide useful spatial and temporal information on the spread of resistance.

## Background

Antimalarial drug resistance is a major concern for malaria control and elimination programmes. Indeed, *Plasmodium falciparum* parasites have consistently developed resistance to the most widely used antimalarials, pushing national malaria control programmes to regular changes in antimalarial drug policy [1]. Artemisinin-based combination therapy (ACT) is now the mainstay for malaria treatment in endemic regions, following recommendations from the World Health Organization (WHO) [2]. However parasites with decreased susceptibility to artemisinin derivatives have emerged over the last ten years in different parts of the Greater Mekong Sub region (GMS) [3–7]. ACTs are failing due to both decreased susceptibility to artemisinin compounds and resistance to their partner drugs in Southeast Asia [8–14]. Strengthening of existing surveillance systems is needed to detect drug resistance in malaria endemic countries as it emerges or spreads to other regions. Antimalarial drug resistance surveillance is currently done through three different strategies: *in vivo* studies such as therapeutic efficacy studies (TESs), *in vitro*/*ex vivo* studies of cultured malaria parasites, and molecular studies assessing known markers of antimalarial drug resistance. These three techniques are complementary, but each has advantages and disadvantages [15]. TES remains the gold standard for informing antimalarial drug policy change, as outcomes have direct clinical relevance [16], but these studies are challenging to conduct due to heavy financial and logistical constraints [17], and they cannot always confirm resistance, especially for combination therapies [18]. Indeed, only monotherapy studies allow for the accurate differentiation of the drug component causing apparent ACT treatment failure [19]. *In vivo*/*ex vivo* studies, such as measurement of IC_50_ (50% inhibitory concentration of a drug) or ring stage survival assays, can provide useful information about parasite susceptibility to antimalarial drugs, but require heavy infrastructure for parasite culture. Performance of these assays is generally restricted to well-equipped laboratories to validate new molecular markers of antimalarial drug resistance [20], or to link a resistance phenotype to a genotype [21]. Molecular studies of antimalarial drug resistance markers provide information about the parasite genetics associated with resistance, *i*.*e*. single nucleotide polymorphisms (SNPs) or gene copy number variations (CNVs) that are associated with decreased susceptibility of parasites to antimalarial drugs. After markers of resistance have been identified by genotype-phenotype discovery studies, detection of these molecular markers provides a feasible means of tracking emergence and/or spread of antimalarial drug resistance, as easy-to-collect dried blood spot (DBS) samples can be used [22,23]. While numerous methodologies for blood spot collection, DNA extraction, PCR amplification, and analysis of molecular markers have been described, standardisation of these approaches is lacking [1]. Given the potential role of molecular surveillance of drug resistance markers, a standardised approach is important to allow for comparability across the globe.

Here we describe the target product profile (TPP), with minimal and optimal characteristics, for techniques to analyse molecular markers associated with antimalarial drug resistance. This TPP was developed by a group of experts from academic institutions, public health institutions and industry at a meeting convened by the Foundation for Innovative New Diagnostics (FIND).

## Methodology

A draft TPP was developed based on a landscape analysis of antimalarial drug resistance surveillance methods performed by FIND [1]. The listed properties were defined according to FIND’s standard procedures (https://www.finddx.org/target-product-profiles/), with characteristics described as either “minimal” or “optimal”. The experts were selected based on their experience and expertise in the field of molecular markers of antimalarial drug resistance. The participants selection was based on a review previously conducted by FIND on the methods used for surveillance of antimalarial drug resistance [1]. Identified experts were contacted by email, invited to participate in the meeting and provided with a brief summary of the meeting’s objectives (S1 Table). Those who confirmed their attendance were provided with the draft TPP prior to the meeting including a questionnaire (S2 Table). The meeting was organised by FIND and held in Geneva on 21 and 22 September 2017 to reach consensus on the TPP. The experts were asked to present the different molecular techniques that are used in their laboratories and discussed their advantages and disadvantages (Table 1).

**Table 1:** Laboratory methods to assess molecular markers associated with antimalarial drug resistance.

Assay	Required equipment and reagents	Required personnel	Assay duration (From DNA extraction to results)	Cost per sample (USD) Excluding labour	Positive and negative controls	Limitations	Appropriate setting for use	Ref.
Mutation-specific-PCR	**Equipment** Incubator Centrifuge Hood Thermocycler Gel electrophoresis unit Gel imaging system Computer **Reagents** DNA extraction reagents PCR reagents	Trained staff	< 8h	8–10	- Parasite DNA sample with known genotype - Sample without DNA template	- Cannot detect copy number variations - Low throughput	- National reference laboratory - Research laboratory	[24–27]
PCR-RFLP	**Equipment** Incubator Centrifuge Hood Thermocycler Gel electrophoresis unit Gel imaging system Computer **Reagents** DNA extraction reagents PCR reagents Restriction enzymes	Trained staff	>24h	7–10	- Parasite DNA sample with known genotype - Sample without DNA template	- Cannot detect copy number variations - Low throughput	- National reference laboratory - Research laboratory	[28,29]
Molecular beacons	**Equipment** Incubator Centrifuge Hood Thermocycler Computer spectrofluorometer **Reagents** DNA extraction reagents PCR reagents Fluorescent oligonucleotide probes	Trained staff	<8h	9–12	- Parasite DNA sample with known genotype - Sample without DNA template	- Cannot detect copy number variations - Low throughput	- National reference laboratory - Research laboratory	[30]
Dot blot hybridization	**Equipment** Equipment Incubator Centrifuge Hood Thermocycler Gel electrophoresis unit Gel imaging system Computer Dot blot unit **Reagents** DNA extraction reagents PCR reagents Dot blot reagents Oligonucleotide probes	Trained staff	>24h	9–12	- Parasite DNA sample with known genotype - Sample without DNA template	- Cannot detect copy number variations - Low throughput	- National reference laboratory - Research laboratory	[31]
Primer extension (Snapshot)	**Equipment** Incubator Centrifuge Hood Thermocycler Gel electrophoresis unit Gel imaging system Computer Sequencer **Reagents** DNA extraction reagents PCR reagents Oligonucleotide probes	Trained staff	>10h	12–15	- Parasite DNA sample with known genotype - Sample without DNA template	- Cannot detect copy number variations - Low throughput	- National reference laboratory - Research laboratory	[32]
Real time PCR	**Equipment** Equipment Incubator Centrifuge Hood Thermocycler Computer **Reagents** DNA extraction reagents PCR reagents Oligonucleotide probes	Trained staff	<6h	13–20	- Parasite DNA sample with known genotype - Sample without DNA template		- National reference laboratory - Research laboratory	[33–35]
Sanger sequencing	**Equipment** Incubator Centrifuge Hood Thermocycler Gel electrophoresis unit Gel imaging system Computer Sequencer **Reagents** PCR reagents Sequencing reagents	Highly trained staff, especially for data analysis	>72h	6–40	- Reference strain	- High initial investment - Requires high volume computing system for data analysis	- Regional reference laboratory - Research laboratory	[36,37]
SSOP-ELISA	**Equipment** Incubator Centrifuge Hood Thermocycler Gel electrophoresis unit Gel imaging system Computer ELISA reader **Reagents** DNA extraction reagents PCR reagents Oligonucleotide probes ELISA plates	Trained staff	<12h	12–14	- Parasite DNA sample with known genotype - Sample without DNA template	- Cannot detect copy number variations - Low throughput	- National reference laboratory - Research laboratory	[38]
Microarray	**Equipment** Incubator Centrifuge Hood Thermocycler Gel electrophoresis unit Gel imaging system Computer Fluorescence scanner **Reagents** DNA extraction reagents PCR reagents Fluorescent oligonucleotide probes Microarray spotted slides	Trained staff	<8h	6–8	- Parasite DNA sample with known genotype - Sample without DNA template	- Cannot detect copy number variations	- National reference laboratory - Research laboratory	[39,40]
Next generation sequencing (WGS, amplicon sequencing)	**Equipment** Incubator Centrifuge Hood Thermocycler Gel electrophoresis unit Gel imaging system Computer Sequencer **Reagents** PCR reagents Sequencing reagents	Highly trained staff, especially for data analysis	>48h	10–200	- Reference strain	- Higher coverage needed to increase specificity - Requires high volume computing system for data analysis	- Regional reference laboratory - Research laboratory	[41,42]
Ligase detection reaction fluorescent microsphere (LDR-FM)	**Equipment** Incubator Centrifuge Hood Thermocycler Gel electrophoresis unit Gel imaging system Computer Magpix instrument **Reagents** DNA extraction reagents PCR reagents Fluorescent oligonucleotide probes	Trained staff	<8h	4–6	- Parasite DNA sample with known genotype - Sample without DNA template	- Cannot detect copy number variations	- National reference laboratory - Research laboratory	[29,43]
Nucleic acid lateral flow immunoassay (NALFIA)	**Equipment** Incubator Centrifuge Hood Thermocycler Gel electrophoresis unit Gel imaging system Computer **Reagents** DNA extraction reagents PCR reagents oligonucleotide probes Lateral flow test	Trained staff	<6h	5–10	- Parasite DNA sample with known genotype - Sample without DNA template	- Cannot detect copy number variations - Low throughput	- National reference laboratory - Research laboratory	[44]
Loop mediated isothermal amplification (LAMP)	**Equipment** Incubator Centrifuge Hood **Reagents** DNA extraction reagents LAMP reagents	Staff with minimal training	<4h	20–120	- Parasite DNA sample with known genotype - Sample without DNA template	- Cannot detect copy number variations - Low throughput>	- Field laboratory	[45,46]
LAMP-lateral flow dipstick	**Equipment** Incubator Centrifuge Hood **Reagents** DNA extraction reagents LAMP reagents Lateral flow test Oligonucleotide probes	Staff with minimal training	<4h	20–120	- Parasite DNA sample with known genotype - Sample without DNA template	- Cannot detect copy number variations - Low throughput	- Field laboratory	[45,47]
MinION	**Equipment** Incubator Centrifuge Hood MinION device **Reagents** DNA extraction reagents MinION reagents	Staff with minimal training for samples analysis Highly trained staff for data analysis	<3days	25–50	- Reference strain>	- High coverage needed to improve specificity	- Field laboratory for sample analysis - National reference laboratory/Research laboratory for data analysis	[48–50]
Q-POC	**Equipment** QPOC device **Reagents** QPOC cassettes Reagents	Staff with minimal training	<30min	TBD	- Parasite DNA sample with known genotype - Sample without DNA template		- Point of care	[51]

A session was organised to go through the draft TPP using the pre-established questionnaire as a guideline. Experts were asked to provide their opinion on the different assay characteristics, and discuss about them to reach a consensus. The discussion was moderated by one of interviewer from FIND. All the final decisions were made by consensus; none of the decisions were taken by voting. Comments and suggestions from the experts were collected and compiled in the meeting’s report. After the meeting, a revised draft TPP following suggestions from the experts’ meeting was sent to the meeting participants along with the meeting’s report. The experts were asked to review the revised draft and the meeting report, and confirm that both documents accurately reflected the discussions they had during the meeting. They were asked as well to provide additional suggestions on the revised TPP, and based on those comments, the TPP was finalised and sent to all participants for final review and approval. More details about the meeting can be found in S3 Table.

## Results

### Participants

Twenty seven experts (including four observers) were invited to the meeting. Eighteen experts (including four observers) were able to attend the meeting, whereas nine experts were not available. All the experts are working in the field of antimalarial drug resistance. The majority of the participants (n = 13 [72.2%]) were research group leaders from academic institutions; other participants were coming from public health institutions such as WHO and the Centers for Disease Control and Prevention (CDC) or industry (Table 2). Most of the participants were coming from institutions based in the United States of America (USA), the United Kingdom (UK), Switzerland and France, while only 7 of them were female (Table 2).

**Table 2:** Participants’ characteristics.

	Number	Percentage (%)
**Affiliation**		
• Academic institutions	13	72.2
• Public Health Institutions /International Organizations	3	16.7
• Industry	2	11.1
**Gender**		
• Female	7	38.9
**Professional qualifications**		
• PhD	10	55.6
• MD & PhD	3	16.7
• MD	3	16.7
• MD & ScD	1	5.6
• ScD	1	5.6
Institutions’ countries*		
USA	5	25
France	4	20
Switzerland	3	15
UK	3	15
Austria	1	5
Denmark	1	5
Kenya	1	5
Netherlands	1	5
Thailand	1	5

*Some participants have a double affiliation.

### General characteristics

#### Intended use

The goal of a molecular assay is to detect genetic markers associated with antimalarial drug resistance in *P*. *falciparum* parasites using blood samples from infected individuals. Discussions were held to assess whether *Plasmodium vivax* should also be included in the TPP. The final consensus was that priority should be given to *P*. *falciparum*, as molecular markers are well characterised for decreased susceptibility to artemisinins and resistance to partner drugs for *P*. *falciparum*, but not for *P*. *vivax*. Rather, currently there is no clear evidence of *P*. *vivax* resistance to artemisinins, and for *P*. *vivax* resistance to chloroquine (CQ), amodiaquine (AQ) and sulfadoxine-pyrimethamine (SP), molecular markers have not been validated [52].

#### Target population

The target population is any individual infected with *P*. *falciparum*.

#### Target users

The target users are highly trained laboratory technicians. There was a consensus that surveillance of antimalarial drug resistance with current technologies would be best conducted by national or regional reference laboratories that receive samples from sentinel sites or other national sources.

#### Implementation level

The target implementation level is regional or national reference laboratories. Having reference laboratories performing all the analyses at a centralised facility will probably be most cost-effective and provide the most accurate results. In addition, constraining the implementation level to reference laboratories simplifies reporting, data monitoring, and procedure harmonization.

### Technical and performance characteristics

The most important performance criteria were analytical sensitivity, analytical specificity, the specific molecular markers to be analysed, test sensitivity, and test specificity (Table 3). Because most samples will come from cross-sectional surveys, the minimum sensitivity for parasitaemia detection was set at the same level as that being used to characterise symptomatic infections. The optimal sensitivity was set to be equivalent to the most sensitive techniques currently used either for molecular diagnosis of malaria or detection of molecular markers associated with antimalarial drug resistance. The consensus about analytical specificity was that the method should be particular for *P*. *falciparum*. As above, it was agreed that molecular markers for *P*. *vivax* resistance are not yet adequately validated. A list of validated *P*. *falciparum* molecular markers was suggested (Table 3). The technique of choice should be able to analyse all relevant molecular markers associated with antimalarial drug resistance. The outcome of the test should be easy to read and interpret (mutant or wild type for SNPs or number of gene copies for CNVs). Optimally, it should be possible to quantify the percentage of each genotype in samples with multiple infections. The sensitivity and specificity of the testing was set to be at least 90% (ideally 95%) compared to Sanger sequencing. The repeatability and reproducibility of the technique were set at kappa >0.8 and >0.7, respectively, for minimal conditions, and >0.9 and >0.8, respectively, for optimal conditions.

**Table 3:** Performance characteristics based on the consensus by the meeting of experts.

Characteristic	Minimal (M)	Optimal (O)	Comment	Ref.
Analytical sensitivity	Limit of detection (LOD) at 200 parasites/μl	Limit of detection at 1 parasite/μl	The optimal analytical sensitivity should be comparable to the sensitivity of Next generation sequencing (NGS) and RT-PCR. The minimal requirement should be the detection of parasites in symptomatic patients	[53,54]
Analytical specificity	Specific for *P*. *falciparum*	Specific for *P*. *falciparum*	*P*. *falciparum* should be prioritized	[55,56]
Molecular markers	*Pfcrt* codon 76 *Pfmdr1* codons 86/1246 and CNV *Pfdhfr* codons 50/51/59/108/164 *Pfdhps* codons 436/437/540/581 *PfKelch-13* codons 446/458/493/539/543/561/580 *Plasmepsin 2/3* CNV *Cytbc1* codon 268	All relevant molecular markers associated with antimalarial drug resistance	*P*. *falciparum* only	
Testing outcome	Binary for SNPs/ number of copies for CNVs	Binary for SNPs with quantification of the different alleles, and number of copies for CNVs	The outcome should be wild type” or “mutant” for each allele, ideally with the concentration of each in mixed infections	[41,53]
Testing sensitivity	> 90% as compared to bi-directional Sanger sequencing	> 95% as compared to bi-directional Sanger sequencing	Sanger sequencing would be used as the gold standard	[41,44]
Testing specificity	> 90% as compared to bi-directional Sanger sequencing	> 95% as compared to bi-directional Sanger sequencing	Same as for sensitivity. However, specificity should be given priority over sensitivity	[41,44]
Repeatability (inter-operators)	Kappa > 0.8	Kappa > 0.9	The technique should be reproducible between technicians.	
Reproducibility (inter-laboratories)	Kappa > 0.7	Kappa > 0.8	The technique should be reproducible between laboratories.	

### Technical and operational characteristics

The operational characteristics of the molecular assay are summarized in Table 4. The discussions during the meeting were mainly on the assay format, assay throughput, and sample matrix. Concerning the assay format, there was consensus that a requirement for use of sophisticated laboratory equipment was appropriate because analyses should be conducted by national or regional reference laboratories. High throughput was preferred; however, it was agreed that the assay should be flexible enough to allow the laboratory to analyse small quantities of samples when appropriate (*i*.*e*. no restriction by batch size). DBS was the preferred format to collect samples. However, good quality filter paper should be used to ensure optimal yield and quality of DNA, especially after long term storage [57]. Optimally, the assay should be able to use DNA extracted from a positive rapid diagnostic test (RDT), as RDTs are currently widely used in malaria endemic countries, especially in Africa, offering at times the best access to samples [58]. Importantly, assays should routinely include negative and positive controls. It is of paramount importance that external controls are included for the assessment of the assay and calibration, and that a good quality control and quality assurance system is implemented to ensure good laboratory practice standardisation.

**Table 4:** Operational characteristics based on the consensus by the meeting of experts.

Operational characteristics				
Characteristic	Minimal (M)	Optimal (O)	Comment	Ref.
Assay format	Lab based equipment at a reference laboratory	Lab based equipment at a reference laboratory		
Assay throughput	High throughput	Automated high throughput	Throughout should be flexible to allow testing of low volumes of samples	
Assay packaging	Standard reagents	Package of single kits with individual reagents sharing user manual	The packaging should be developed for a high throughput assay	
Operation conditions	15°C to 30°C [Up to 60% relative humidity (RH)]	15°C to 35°C [Up to 80% RH]	The assay should be developed to work in a reference laboratory in a malaria-endemic country	
Reagents transportation and storage stability	Cold chain	Cold chain	Cold chain is acceptable as the assay would be developed for reference laboratories	
In use stability	4 hours at 15°C to 30°C [Up to 60% RH]	4 hours at 15°C to 35°C [Up to 80% RH]	Once reagents have been prepared, they should be stable in a reference laboratory	
Reagents reconstitution	All reagents ready to use	All reagents ready to use		
Equipment	Hoods/Thermocycler/ sequencer/Computer/Gel electrophoresis unit/Gel imaging system/Other equipment	Hoods/Thermocycler/ sequencer/Computer/ Gel electrophoresis unit/Gel imaging system/other equipment	For reference laboratories, different equipment could be used	
Power requirement	Electric	Electric	The equipment needs to be at least electric operated (M) or have a battery to be used in places where power cuts could be frequent (O)	
Maintenance	Every 6 months	Once a year	Regular maintenance should be possible in reference laboratories	
Sample type	Finger stick blood	Finger stick blood		
Sample matrix	Dried blood spot (DBS)	Used RDT	DBS should be the default matrix for samples collection, and ideally used RDT should be used as source of DNA	
Sample preparation	≤ 5 steps	≤ 3		
Overall test preparation	≤ 10 steps, of which ≤2 are timed	≤ 3 steps, of which ≤1 are timed	Same as above	
Time to results	1 months	1 week	From sample collection to results	
Internal control	Included	Included	Both negative and positive controls should be included with all assays.	
External control	Available	Included	Both negative and positive controls should be included with all assays.	
Assay interpretation	Unambiguous, recorded by operator	Unambiguous, recorded by operator or electronically	The interpretation of the results should be simple	
Data capture	Manual by operator	Electronic automated	Data capture should be flexible and adaptable	
Data transfer	Manual by operator	Automated via internet or Global System for Mobile Communications (GSM) connectivity	Same as above for data transfer	
Training	≤ 1 week for technician with little experience	≤ 3 days for technician with little experience	The technique should be easy to learn	
Biosafety	Moderate individual and low public health risk	Low individual and public health risk	According to risk-based classification of diagnostics for WHO prequalification	[59]

### Assay cost characteristics

The cost of the assay should be low enough to be affordable in developing countries. The cost to analyse one sample for all mutations should ideally not be more than 10 USD, comparable to or cheaper than widely used PCR-RFLP assays [29].

## Discussion

Molecular markers of antimalarial drug resistance have proved to be useful for detection of early resistance emergence [5,7,60], spread of resistance [61], or absence of resistance [62], and are easy to interpret [63]. Although TESs provide valuable resistance measurements that are easiest to directly translate to policy, they are confounded by many factors, including clinical immunity and varied pharmacokinetics, and they require extensive time for completion, so resistance may only be apparent once parasites resistant to both components of a drug combination have spread widely [64]. Molecular techniques have the advantage of providing information in real time about the prevalence and ideally the frequency of resistant parasite strains circulating in the population using easily collected DBS or RDT samples [42,58,65], and this information is not typically confounded by clinical immunity. Even though, the presence of resistant parasites does not necessarily predict treatment failure [66], increasing prevalence of validated molecular markers of antimalarial drug resistance is associated with increasing treatment failure, and thus molecular markers offer a valuable early indicator of resistance emergence [67], and a practicable means of determining thresholds for policy makers. As an example, the WHO policy on Intermittent preventive treatment for infants (IPTi) with SP recommends ≤50% prevalence of *Pfdhps* 540 mutation as the threshold for implementation of SP-IPTi [68]. A variety of different techniques to assess molecular markers associated with antimalarial drug resistance are already available (Table 1), however standardisation is needed to improve the quality of generated data [1].

New and improved technologies should focus on simple techniques that can be used by laboratories in malaria endemic countries. Techniques should be highly sensitive to detect minority strains, but also highly specific to yield accurate results. Indeed, according to the consensus obtained during the meeting of experts, priority should be given to specificity over sensitivity; it is better to miss strains at low level than to give inaccurate prevalence data. Increased multiplicity of infection in high transmission settings may compromise assessment of antimalarial drug resistance molecular markers [69]. Indeed, genotyping of samples with multiple infections is challenging, as it is difficult to link different mutations to a specific strain, and therefore accurately assess haplotypes or frequencies of specific strains, in particular when considering CNV. New technologies under development, including amplicon sequencing, may allow assessment of drug resistance variants among polygenomic infections [70–74]. However, in the setting of high multiplicity of infection, prevalence data remains useful for surveillance purposes [75]. Determination of CNV is a minimal requirement in this TPP, as resistance to some of the important artemisinin partner drugs such as mefloquine and piperaquine is associated with changes in gene copy numbers [76,77]. Currently, sequencing technologies and real-time PCR offer most of the desired characteristics described in the current TPPs, including the determination of CNVs (Table 1), and those technologies are becoming increasingly available and affordable in developing countries [1]. Other new techniques are in development that could improve standardisation, with no DNA amplification [78,79] or DNA extraction step requirement [51,80]. However, these techniques are still at an early stage of development and are mainly under evaluation for diagnosis, and not surveillance.

Recent advances in sequencing technologies, such as next-generation sequencing (NGS) platforms that enable rapid whole genome sequencing (WGS), can provide in-depth information about molecular determinants of resistance, allowing detailed assessment of the spread of resistant strains [81–83]. They can provide as well information about new emerging mutations before they can be confirmed by phenotypic data from *in vitro* assessments and clinical data when available. The main objective of a molecular-based surveillance system should be the detection of resistance before it spreads. For artemisinin resistance, different foci have been discovered, and molecular determinants other than *pfKelch13* may be involved [84,85], requiring a continuous search and validation for new molecular markers. The development of a surveillance system included in the local health system could be envisioned; samples would be collected at health posts, centres or hospitals and sent to reference laboratories for analysis and validation, while clinical data could be shared through electronic-based information system [86]. Combined with local epidemiological data; drug usage and treatment efficacy data, WGS data could provide valuable information for modelling and predicting the spread of antimalarial drug resistance [87]. The recent development of MinION nanopore portable sequencer and its application to molecular markers of resistance could facilitate as well sample analysis at point of care, while the data analysis could still be performed in the central reference laboratory [48,50]. NGS technologies also allow pooling of different samples by indexing them to reduce the analysis costs [41,42]. Even though the costs of all these NGS technologies have dramatically reduced in recent years and are affordable for developing countries, they still require high expertise in data analysis, and high computing power that are not always available in those countries. However, the establishment of centres of excellence or regional reference laboratories could overcome this issue.

To ensure the accuracy and the comparability of the results from different laboratories, a good external quality assurance (EQA) system should be implemented, providing validated and standardised external control material [88,89]. Indeed, different laboratories may use different protocols and standard operating procedures (SOPs) for the same methodology, and there is variability in operating procedures in different laboratories. An analogous EQA scheme for malaria nucleic acid amplification testing external quality assurance (NAAT EQA) has been developed by WHO and FIND [90], and could potentially be expanded to molecular markers of resistance.

## Conclusion

In summary, techniques already exist with most of the required characteristics in this TPP for assays to analyse molecular markers associated with antimalarial drug resistance, and could be rapidly implemented in reference laboratories. Other techniques in development fulfil most of the criteria specified by the TPP and could potentially improve data analysis standardisation. However, the implementation of different techniques for routine surveillance of antimalarial drug resistance would need a consensus from policy makers to define implementation procedures, optimise their use, and implement good EQA practices. This TPP can also be used by assay manufacturers to guide development of new technologies to facilitate efficient surveillance of molecular markers associated with antimalarial drug resistance in endemic settings.

## Supporting information

S1 Table List of invited experts. (DOCX)

S2 Table Draft target product profile and pre-meeting questionnaire. (PDF)

S3 Table COREQ checklist. (DOCX)
