# Supplementary material for: Molecular assays for antimalarial drug resistance surveillance: A target product profile
Source: PLoS One. 2018 Sep 20;13(9):e0204347. doi: 10.1371/journal.pone.0204347 (PMC6147503; doi:10.1371/journal.pone.0204347)
Supplement: S1 Table — (DOCX) [file pone.0204347.s001.docx]

| **Names** | **Institution (s)** | **City/Country** | | | **Attended** | **Reason for not attending** |
| --- | --- | --- | --- | --- | --- | --- |
| Frederic Ariey | INSERM 1016, Institut Cochin, Université Paris Descartes  Service de Parasitologie-Mycologie, Hôpital Cochin | Paris/France | | | yes |  |
| Michal Alifrangis | Centre for Medical Parasitology, Department of Immunology and Microbiology, University of Copenhagen  Department of Infectious Disease, Copenhagen University Hospital | Copenhagen/Denmark | | | No | Busy schedule |
| Hans-Peter Beck | Medical Parasitology and Infection Biology department, Swiss Tropical and Public Health Institute  University of Basel | Basel/Switzerland | | | yes |  |
| Souleymane Dama | Malaria Research & Training Center, Faculty of Pharmacy and  Faculty of Medicine and Dentistry, University of Sciences  Techniques and Technologies of Bamako | Bamako/Mali | | | No | Not able to get visa on time |
| Abdoulaye Djimde | Malaria Research & Training Center, Faculty of Pharmacy and  Faculty of Medicine and Dentistry, University of Sciences  Techniques and Technologies of Bamako | Bamako/Mali | | | No | Not able to get visa on time |
| David A. Fidock | Department of Microbiology and Immunology, Columbia University Medical Center  Division of Infectious Diseases, Department of Medicine, Columbia University Medical Center | New York/USA | | | No | Busy schedule |
| Edwin Kamau | Walter Reed Army Institute of Research  KEMRI/United States Army Medical Research Unit-Kenya | Silver Spring/USA  Kisumu/Kenya | | | yes |  |
| Sanjeev Krishna | Institute for Infection & Immunity, St George's University of London | London/UK | | | yes |  |
| Miriam Laufer | Division of Malaria Research, Institute for Global Health, University of Maryland School of Medicine | Baltimore/USA | | | No | Busy schedule |
| Eric Legrand | Malaria Genetic and Resistance Group, Biology of Host-Parasite Interactions Unit, Institut Pasteur | Paris/France | | | yes |  |
| Naomi Lucchi | Malaria Branch, Division of Parasitic Diseases and Malaria, Center for Global Health, Centers for Disease Control and Prevention | Atlanta/USA | | | yes |  |
| Didier Menard | Malaria Genetics and Resistance Group, Institut Pasteur | Paris/France | | | No | Conflicting meeting |
| Olivo Miotto | Mahidol-Oxford Research Unit, Faculty of Tropical Medicine, Mahidol University  Big Data Institute, Li Ka Shing Centre for Health Information and Discovery, Old Road Campus, Oxford  Wellcome Trust Sanger Institute | Bangkok/Thailand  Oxford/UK  Hinxton/UK | | | yes |  |
| Sidsel Nag | Centre for Medical Parasitology, Department of Immunology and Microbiology, University of Copenhagen  Department of Infectious Disease, Copenhagen University Hospital | Copenhagen/Denmark | | | yes |  |
| Harald Noedl | Institute of Specific Prophylaxis and Tropical Medicine, Medical University of Vienna | Vienna/Austria | | | yes |  |
| Christopher V. Plowe | Division of Malaria Research, Institute for Global Health, University of Maryland School of Medicine | Baltimore/USA | | | No | Conflicting meeting |
| Cally Roper | Faculty of Infectious and Tropical Diseases, London School of Hygiene and Tropical Medicine | London/UK | | | yes |  |
| Philip J. Rosenthal | Department of Medicine, University of California San Francisco | San Francisco/USA | | | yes |  |
| Henk DFH Schallig | Department of Medical Microbiology, Academic Medical Centre | Amsterdam/The Netherlands | | | yes |  |
| Colin J. Sutherland | Department of Immunology & Infection, London School of Hygiene and Tropical Medicine | London/UK | | | No | On sabbatical leave |
| Steve M. Taylor | Division of Infectious Diseases and Duke Global Health Institute, Duke University Medical Center | Durham/USA | | | yes |  |
| Venkatachalam Udhayakumar | Malaria Branch, Division of Parasitic Diseases and Malaria, Center for Global Health, Centers for Disease Control and Prevention | Atlanta/USA | | | No | Busy schedule |
| Sarah K. Volkman | Department of Immunology and Infectious Diseases, Harvard T. H. Chan School of Public Health  School of Nursing and Health Sciences, Simmons College  Infectious Disease Initiative, Broad Institute of MIT and Harvard | Boston/USA  Cambridge/USA | | | yes |  |
|  | | | | | | |
| **Observers** | **Institution (s)** | | **Country** | **Attended** | | **Reason for not attending** |
| Jane A. Cunningham | Global Malaria Programme, World Health Organization | | Geneva/  Switzerland | Yes  (only day 2) | |  |
| Laurence Ganée | Biomerieux | | Marcy l’étoile/ France | yes | |  |
| Karine Kaiser | Biomerieux | | Marcy l’étoile/ France | yes | |  |
| Pascal Ringwald | Global Malaria Programme, World Health Organization | | Geneva/ Switzerland | Yes  (only day 1) | |  |
