# Supplementary material for: Molecular assays for antimalarial drug resistance surveillance: A target product profile
Source: PLoS One. 2018 Sep 20;13(9):e0204347. doi: 10.1371/journal.pone.0204347 (PMC6147503; doi:10.1371/journal.pone.0204347)
Supplement: S2 Table — (PDF) [file pone.0204347.s002.pdf]

| DRAFT TARGET PRODUCT PROFILE | TPP molecular markers<br>Antimalarial drug resistance surveillance |
|---|---|
| Version 1.0 | |
| Release date: | |

## Introduction

This document is a working draft intended to the communication between experts on molecular markers of antimalarial drug resistance and FIND to inform and finalise a target product profile for a test for the detection of molecular markers associated with antimalarial drug resistance. The final version of this document will be publicly available and will serve to inform developers and guide product development.

This TPP is divided in sections covering scope, performance, operational aspects, cost and other characteristics. References, including modelling data when available, supporting suggested characteristic target values are indicated.

This document was drafted by FIND (Christian Nsanzabana and Iveth J Gonzalez).

## Definitions

Characteristic

Refers to a specific requirement or a specification that is measurable.

Minimal

Refers to the lowest acceptable output for that characteristic for a satisfactory technique.

Optimal

Refers to the lowest acceptable output for that characteristic for an ideal technique. Meeting the optimal characteristics provides the greatest differentiation and the greatest impact of end users, clinicians and patients. Developers would ideally design and develop their solutions to meet the optimal characteristics in all cases.

## Abbreviations

M Minimal
O Optimal
p/µl parasites/microliter
RH Relative humidity
TPP Target product profile
WHO World Health Organization

| Scope | |
|---|---|
| Key indicators and existing recommendations | • Antimalarial drug treatment policy change is based on results from Therapeutic efficacy studies (TES) [1]<br>• Molecular markers are used for surveillance and to provide information about the extent of antimalarial drug resistance |
| Rationale | Currently, surveillance of antimalarial drug resistance is conducted through three different approaches: (1) therapeutic efficacy studies to assess the efficacy of drugs in patients; (2) *in vitro*/*ex vivo* studies to evaluate parasite susceptibility to the drugs; or (3) molecular studies to identify new or detect known gene mutations and/or copy number changes that have been associated with drug resistance. These methods are complementary, as they evaluate different aspects of drug resistance. From the three approaches, molecular markers surveillance is probably the most appropriate and cost-effective way for continuous surveillance as it can give information about the prevalence of resistant parasites before this is reflected in treatment failures, giving the possibility to policy makers to confirm resistance with therapeutic efficacy studies, plan and promptly implement changes in treatment guidelines. The technology landscape for molecular analysis of drug resistance genes is changing quite rapidly, and new more affordable techniques are becoming available. They also offer the advantage of high throughput analysis. Many molecular techniques have been developed to assess the molecular markers associated with drug resistance, but none of them was really developed specifically for antimalarial drug resistance. This TPP will help experts and developers to guide the development of new techniques or to optimise the existing techniques. |
| Intended use | The goal is to detect molecular markers associated with antimalarial drug resistance in *Plasmodium falciparum* parasites using blood samples from infected individuals. |
| Target population | The target population is any individual infected with *P. falciparum* |
| Target users | The target users are trained laboratory technicians. |
| Implementation level | The target implementation levels are health centres or national reference laboratories. |

| Performance characteristics | | | | |
|---|---|---|---|---|
| Characteristic | Minimal (M) | Optimal (O) | Comment | Ref. |
| Analytical sensitivity | Limit of detection at 1 pg of DNA | Limit of detection at $10^{-5}$ ng of DNA | The analytical sensitivity should be comparable to the sensitivity of NGS or RT-PCR | [2,3] |
| Analytical specificity | Specific for *P. falciparum*, do not cross-react with any other specie or human genes | Specific for *P. falciparum*, do not cross-react with any other specie or human genes | *P. falciparum* is prioritized due to the potential development of resistance to ACTs | [4,5] |

DRAFT

DRAFT

| | | | | |
|---|---|---|---|---|
| Testing outcome | Binary | Binary with quantification of the different alleles | The outcome should be “wild type” or “mutant” for each allele, and for “O” it should give the concentration of each in mixed infections | [2,6] |
| Testing sensitivity | > 95% as compared to sanger bi-directional sequencing | > 99% as compared to sanger bi-directional sequencing | Sanger sequencing would be used as the gold standard | [6,7] |
| Testing specificity | > 95% as compared to sanger bi-directional sequencing | > 99% as compared to sanger bi-directional sequencing | Same as for sensitivity | [6,7] |
| Repeatability (inter-operators) | Kappa > 0.8 | Kappa > 0.9 | The technique should be used by different technicians in the same laboratory and still provides reliable results | |
| Reproducibility (inter-laboratories) | Kappa > 0.7 | Kappa > 0.8 | As the technique would be used in different laboratories in different countries, good reproducibility should ensure a better standardisation of the data | |
| **Operational characteristics** | | | | |
| Characteristic | Minimal (M) | Optimal (O) | Comment | Ref. |
| Assay format | Lab based equipment | health post based equipment | The assay should be mainly used by reference laboratories for surveillance | |
| Assay throughput | High throughput (96 well plates format) | Automated high throughput(96 | As this is for surveillance, this needs to be a high | |

| | | | | |
|---|---|---|---|---|
| | | well plates format) | throughput assay to analyse multiple samples simultaneously | |
| Assay packaging | Package of single kits sharing reagents and user manual | Package of single kits with individual reagents sharing user manual | The packaging should be developed for a high throughput assay | |
| Operation conditions | 15°C to 30°C (Up to 60% relative humidity /RH) | 15°C to 35°C Up to 80% RH | The assay should be developed to work in a reference laboratory (M) or a health post (O) | |
| Transportation and storage stability | Cold chain | ≥12 months at 35°C and 70% RH with transport stress (3 days at 60°C) no cold chain needed | If the assay is developed for reference laboratories, cold chain would be acceptable, however for a health post or clinic, it should be stable at ambient temperature | |
| In use stability | 4 hours | < 30 minutes | The time to results are defined for a laboratory based equipment (M) and for a health post/clinic based equipment (O) | |
| Reagents reconstitution | Reconstitution of reagents takes < 60 min | All reagents ready to use | For reference laboratories, reagents can be reconstituted (M), but for health post/clinics it is preferable to have reagents ready to use | |
| Equipment | Thermocycler/portable sequencer/computer | One device | For reference laboratories, different | |

DRAFT

| | | | | |
|---|---|---|---|---|
| | | | equipment could be used, however for health post and/or clinics one device that performs all the different steps would be preferable | |
| Power requirement | Electric | Battery operated with ≥ 24h autonomy | The equipment needs to be at least electric operated (M) or have a battery to be used in places where power cuts could be frequent (O) | |
| Maintenance | Every 6 months | Once a year | Regular maintenance is possible in reference laboratories, but would be more difficult in more remote areas | |
| Sample type | Capillary blood | Capillary blood | Capillary blood is the sample of choice for studying malaria parasites | |
| Sample preparation | ≤ 5 steps | None | To avoid cross contamination and mixing of samples, very few steps should be used for the sample preparation | |
| Overall test preparation | ≤ 10 steps, of which ≤2 are timed | ≤ 3 steps, of which ≤1 are timed | Same as above | |
| Time to results | ≤ 4 hours | ≤ 30 min | From sample collection to final results | |
| Internal control | Included | Included | Internal control should be | |

FIND Because diagnosis matters

| | | | | |
|---|---|---|---|---|
| | | | available to confirm I if the detection of molecular markers has worked properly | |
| External control | Available | Included | External control should be available as well for calibration | |
| Assay interpretation | Univocal, recorded by operator | Univocal, recorded by operator or electronically | The interpretation of the results should be simplified to avoid misinterpretation, and allow easy transfer of the data | |
| Data capture | Manual by operator | Electronic automated | Automated data capture would decrease the error rate | |
| Data transfer | Manual by operator | Automated via internet or GSM connectivity | Same as above for data transfer | |
| Training | ≤ 1 week for technician with little experience | ≤ 3 days for technician or health worker with little experience | The technique should be easy to learn for unexperienced lab technicians to make sure the change of technicians in a laboratory does not affect the quality of the results | |
| Biosafety | Moderate individual and low public health risk | Low individual and public health risk | According to risk-based classification of diagnostics for WHO prequalification | [8] |
| **Cost characteristics** | | | | |
| Characteristic | Minimal (M) | Optimal (O) | Comment | Ref. |

DRAFT

| | | | | |
|---|---|---|---|---|
| Cost of analysis | ≤ 50 USD per sample | ≤ 10 USD per sample | Cost not including labour | [6] |
| **Miscellaneous characteristics** | | | | |
| Characteristic | Minimal (M) | Optimal (O) | Comment | Ref. |
| Language | English | English, Spanish, French, Portuguese | Ideally it should be available in different languages to avoid misunderstanding in the protocol, however, by defaults the protocol should be available in English | |

**Specific questions**

1. What are currently the most important limitations for the effective detection of molecular markers associated with antimalarial resistance?

2. In light of these, please review the draft TPP and discuss the following points:

    a. Are the proposed analytical sensitivity and specificity reasonable? If not, could you please propose values?

    b. Are the proposed diagnostic sensitivity and specificity reasonable? If not could you please propose values?

    c. Do you agree with the proposed stability for operation conditions and transport storage? If not, please propose values

3. Is there any other characteristic in the TPP that you would like to discuss further?

4. Could you please also advise on the best options for samples collection, storage and processing?

5. Do you see any problem with data interpretation? If yes, what would you advise to solve the issue?

**References**

1. WHO. METHODS FOR SURVEILLANCE OF ANTIMALARIAL DRUG EFFICACY [Internet]. Geneva; 2009. Available from: http://apps.who.int/iris/bitstream/10665/44048/1/9789241597531_eng.pdf

2. Daniels R, Ndiaye D, Wall M, McKinney J, Séne PD, Sabeti PC, et al. Rapid, field-deployable method for genotyping and discovery of single-nucleotide polymorphisms associated with drug resistance in Plasmodium falciparum. Antimicrob. Agents Chemother. [Internet]. 2012 [cited 2017 Sep 5];56:2976–86. Available from: http://aac.asm.org/cgi/doi/10.1128/AAC.05737-11

3. Robin JD, Ludlow AT, LaRanger R, Wright WE, Shay JW. Comparison of DNA Quantification Methods for Next Generation Sequencing. Sci. Rep. [Internet]. Nature Publishing Group; 2016 [cited 2017 Sep 11];6:24067. Available from: http://www.ncbi.nlm.nih.gov/pubmed/27048884

4. WHO. Global plan for artemisinin resistance containment (GPARC) [Internet]. Geneva; 2011. Available from: http://apps.who.int/iris/bitstream/10665/44482/1/9789241500838_eng.pdf?ua=1

5. WHO. Minutes of the Evidence Review Group meeting on the emergence and spread of multidrug- resistant Plasmodium falciparum lineages in the Greater Mekong subregion [Internet]. Geneva; 2016. Available from: http://www.who.int/malaria/mpac/mpac-mar2017-erg-multidrug-resistance-session6.pdf

6. Taylor SM, Parobek CM, Aragam N, Ngasala BE, Mårtensson A, Meshnick SR, et al. Pooled deep sequencing of Plasmodium falciparum isolates: an efficient and scalable tool to quantify prevailing malaria drug-resistance genotypes. J. Infect. Dis. [Internet]. 2013 [cited 2017 Apr 6];208:1998–2006. Available from: https://academic.oup.com/jid/article-lookup/doi/10.1093/infdis/jit392

7. Moers APHA, Hallett RL, Burrow R, Schallig HDFH, Sutherland CJ, van Amerongen A. Detection of single-nucleotide polymorphisms in Plasmodium falciparum by PCR primer extension and lateral flow immunoassay. Antimicrob. Agents Chemother. [Internet]. 2015 [cited 2017 Apr 6];59:365–71. Available from: http://aac.asm.org/lookup/doi/10.1128/AAC.03395-14

8. WHO/PQDx. A RISK BASED APPROACH FOR THE ASSESSMENT OF IN VITRO DIAGNOSTICS (IVDs) [Internet]. Geneva; 2014. Available from: http://www.who.int/diagnostics_laboratory/evaluations/140513_risk_based_assessment_approach_buffet.pdf?ua=1

DRAFT
