# Supplementary material for: Molecular assays for antimalarial drug resistance surveillance: A target product profile
Source: PLoS One. 2018 Sep 20;13(9):e0204347. doi: 10.1371/journal.pone.0204347 (PMC6147503; doi:10.1371/journal.pone.0204347)
Supplement: S3 Table — (DOCX) [file pone.0204347.s003.docx]

| **ITEM** | **RESPONSE** |
| --- | --- |
| **Domain 1: Research team and reflexivity** | |
| 1. Interviewers/facilitators | Iveth J. Gonzalez; Xavier C. Ding and Christian Nsanzabana |
| 1. Credentials | Iveth J. Gonzalez, MD, PhD  Xavier C. Ding, PhD  Christian Nsanzabana, PhD |
| 1. Occupation | Iveth J. Gonzalez: Head of the Malaria Programme; Foundation for Innovative New Diagnostics (FIND)  Xavier C. Ding: Senior scientific officer, FIND  Christian Nsanzabana, Scientific officer, FIND |
| 1. Gender | Iveth J. Gonzalez: female  Xavier Ding: male  Christian Nsanzabana: male |
| 1. Experience and training | - Christian Nsanzabana has been working on malaria for more than 10 years. He is a biologist by training, and holds a PhD in Life Sciences from the University of Neuchâtel, Switzerland. He has been working on different aspects of malaria, including the assessment of antimalarial drug resistance molecular markers and their association with clinical outcomes, quality control of improved malaria diagnostics and vector control. - Xavier C. Ding holds a PhD in Molecular Biology from the University of Basel, Switzerland. He has been working on different aspects of malaria over the last ten years, going from drug discovery to development of new malaria diagnostic tests. - Iveth J. Gonzalez has more than 15 years of experience in basic and clinical research applied to tropical diseases, mainly malaria, leishmaniasis, and Chagas disease. Her malaria research focused on vaccine development, identification of clinical and molecular factors associated with antimalarial drug resistance, and development, implementation, and quality control of improved diagnostics for malaria control and elimination. She holds a Medical degree from the Universidad del Valle, Colombia and a PhD in Life Sciences from the University of Lausanne, Switzerland. |
| 1. Relationship established | Some of the study participants were collaborating with FIND prior to the meeting. |
| 1. Participant knowledge of the interviewers | Most of the participants knew the interviewers through different collaborative projects that have been conducted by FIND and other institutions. Two of the participants are previous mentors for Christian Nsanzabana. |
| 1. Interviewers’ characteristics | The interviewers were employees from FIND. FIND was engaged in a process to develop a strategic plan to work on technology development for assessing antimalarial drug resistance. |
| **Domain 2: Study design** | |
| 1. Methodological orientation and theory | Malaria has dramatically decreased over the last 15 years, but the gains are jeopardised with the development of resistance to antimalarial drugs. There is a need to strengthen existing surveillance systems to better respond to this threat. Surveillance using molecular markers of antimalarial drug resistance is probably the most cost effective way that can provide early information on the development of resistance. Therefore a meeting of experts on this topic has been organised to review the landscape of technologies available for molecular markers assessment, design a proficiency testing programme and a target product profile for to support the development of improved tools to assess molecular markers for surveillance of antimalarial drug resistance. |
| 1. sampling | Participants were selected based on their experience in the field of molecular markers of antimalarial drug resistance, and their publication records on the topic. Previous to the meeting a landscape analysis was conducted by FIND on the different tools used to assess antimalarial drug resistance: *Nsanzabana C, Djalle D, Guérin PJ, Ménard D, González IJ. Tools for surveillance of anti-malarial drug resistance: an assessment of the current landscape. Malar J. 2018; 17(1):75.* Experts identified in the landscape analysis were invited for the meeting. |
| 1. Method of approach | Participants were approached by email. |
| 1. Sample size | Eighteen experts including 4 observers |
| 1. Non-participation | Nine experts were not able to attend the meeting due to conflicting meetings or were not able to get visa on time to travel to the meeting venue. |
| 1. Setting of data collection | A meeting with the experts was organised in a hotel in Geneva, Switzerland. |
| 1. Presence of non-participants | Four observers attended the meeting: two from the World Health Organization and two from the industry. |
| 1. Description of sample | **Qualifications:**   - PhD: 10 - MD & PhD: 3 - MD: 3 - MD&ScD: 1 - Sc.D: 1   **Type of institution:**   - Academic: 13 - Public Health Institutions/International Organisations:3 - Industry: 2   **Gender:**   - Male: 11 - Female: 7   **Institutions’ countries*:**   - USA: 5 - France: 4 - UK: 3 - Switzerland: 3 - Netherlands: 1 - Denmark: 1 - Kenya: 1 - Austria: 1 - Thailand: 1   *Some participants have a double affiliation |
| 1. Interview guide | Questions were provided in advance (2 weeks prior to the meeting) to the experts by the interviewers. |
| 1. Repeat interviews | There were no repeat interview |
| 1. Audio/visual recording | No audio or visual recording was performed |
| 1. Field notes | Notes were taken by the interviewers during the meeting. |
| 1. Duration | The duration of the meeting was 1.5 day. |
| 1. Data saturation | Data saturation was not discussed |
| 1. Transcripts returned | A draft meeting report was shared with the meeting participants and they had the opportunity to comment and make correction if need be. |
| **Domain 3: analysis and findings** | |
| 1. Number of data coders | Data form the interviews were not transformed or coded. |
| 1. Description of the coding tree | Not applicable |
| 1. Derivation of themes | The theme was identified in advance by the interviewers, and participants were selected based on the theme and their experience. |
| 1. Software | Not software was used to manage the data. |
| 1. Participant checking | Participants had the opportunity to review and comment on the meeting report and the manuscript. |
| 1. Quotations presented | A summary of each participant presentation during the meeting was provided in the meeting report. |
| 1. Data and findings consistent | The data presented are supporting the conclusions of the meeting. |
| 1. Clarity of major themes | Major themes were clearly presented in the findings. |
| 1. Clarity of minor themes | Diverse cases and minor themes are clearly presented in the meeting report. |
